# Enhancing public health research: a viewpoint report on the transition to secure, cloud-based systems

**DOI:** 10.3389/fpubh.2023.1270450

**Published:** 2024-01-08

**Authors:** Plinio Pelegrini Morita, Jasleen Kaur, Pedro Augusto Da Silva E Souza Miranda

**Affiliations:** ^1^School of Public Health Sciences, University of Waterloo, Waterloo, ON, Canada; ^2^Department of Systems Design Engineering, University of Waterloo, Waterloo, ON, Canada; ^3^Research Institute for Aging, University of Waterloo, Waterloo, ON, Canada; ^4^Centre for Digital Therapeutics, Techna Institute, University Health Network, Toronto, ON, Canada; ^5^Institute of Health Policy, Management, and Evaluation, Dalla Lana School of Public Health, University of Toronto, Toronto, ON, Canada

**Keywords:** UbiLab research environment, public health research, Personal Health Information (PHI), data governance in healthcare, cybersecurity standards for PHI, azure cloud-based research

## 1 Introduction

From smart devices to smart home technologies, Personal Health Information (PHI) is being collected on a previously unprecedented level (1–8). The individual and population metrics gathered can transform public health research, providing valuable insights into population health, disease trends, and effective interventions. Despite advancements in data availability, collection, and analysis (9–13), the use of PHI for research has been hindered by storage, cybersecurity, and data governance challenges (6–8, 14–18). PHI has traditionally been stored in local databases or filesystems which lack sufficient cybersecurity and data governance. This leaves sensitive health information vulnerable to unauthorized access and malicious attacks (3, 5, 19–28). Local databases also lack scalability, making it difficult to accommodate large volumes of data and perform computationally intensive tasks (10, 29–32).

Cloud-based solutions have emerged to address these challenges (33). Our rapid literature review (34–39) identified several frameworks such as InfusedHeart (34), I-Health (38), and Blockchain-Based Personalized Federated Learning (39), which leverage cloud computing for public health applications. While these solutions offer insights into the potential of cloud services, it's crucial to note that their compliance with healthcare standards such as PIPEDA (17), HIPAA (18), and GDPR (15) varies. Some may partially meet these standards, addressing certain aspects of Personal Health Information (PHI) management, but there remains a lack of a comprehensive solution fully aligned with all these regulatory requirements. This gap underscores the need for a tailored approach, such as the UbiSECE framework, which is specifically designed to address the complex requirements of PHI in public health research, ensuring full compliance with these critical healthcare standards.

Microsoft Azure (33, 40), a leading cloud platform, has gained popularity in public health research due to its robust infrastructure and compliance with industry standards (41). The Ubiquitous health technology lab (UbiLab) at the University of Waterloo has faced and addressed the challenges associated with the use of PHI for public health research (42). This paper aimed to share our experiences and insights gained in the adoption of UbiSECE, a cloud-based data governance framework. UbiSECE is based on Microsoft Azure's governance architecture guidelines and incorporates NIST 800–53 compliance with healthcare standards such as PIPEDA, HIPAA, and GDPR (15, 17, 18, 40, 41). It also implements role-based access controls and centralizes data storage. The framework shared here serves as a blueprint for the field of public health research to create streamlined and efficient platforms for managing PHI. To assist readers, a Glossary of specialized terms and acronyms used throughout this paper, such as PHI, PIPEDA, HIPAA, GDPR, NIST and others, is provided at the end of the document. This Glossary aims to clarify key concepts and ensure a clear understanding of the technical aspects discussed.

## 2 Phases

### 2.1 Phase 1- local system

#### 2.1.1 Scenario and benefits

In this initial phase, each UbiLab researcher operated independently, using their own system for research data and resources. This approach resulted in a spread of data across individual computers with minimal centralized storage. Despite the challenges this posed, there were implicit benefits in this setup. Researchers experienced a certain level of comfort and familiarity with their own systems, which might have allowed for ease of use and adaptability to individual working styles. Furthermore, this decentralized approach could have been perceived as more cost-effective initially, as it relied on existing resources without additional investment in centralized infrastructure.

#### 2.1.2 Challenges

The limited utilization of cloud resources and data sharing created a fragmented landscape of resources, often leading to a “sandbox” effect between projects. This phase was marked by a lack of standardized data storage solutions, such as SQL or JSON databases, and an absence of unified data governance frameworks. Cybersecurity measures were not adequately established, leaving sensitive data potentially vulnerable. Additionally, the management of credentials was limited and primarily facilitated by the university's Information Systems & Technology (IST) department, indicating a reliance on external support for essential security processes. There was also a notable deficiency in the IT infrastructure necessary for effectively managing study participants' informed consent and re-consent, which are critical components of ethical research practices. Moreover, the detailed management of data processing costs was inefficient, leading to potential resource wastage and budgetary concerns.

### 2.2 Phase 2- UbiLab azure general environment

#### 2.2.1 Scenario and benefits

In Phase 2, the UbiLab research team made a significant leap by upgrading to a unified cloud-based research environment utilizing Microsoft Azure. This strategic shift enabled the centralization of data storage and sharing within individual research project groups. Additionally, the team implemented enhanced data governance mechanisms, marking a pivotal change in the management and accessibility of research data.

The transition to a cloud-based architecture brought about several key benefits. Firstly, it facilitated improved access to Personal Health Information (PHI) and the utilization of big data, which are crucial for advanced public health research. Secondly, the cloud environment simplified collaboration with third parties and industry partners, making the sharing and analysis of data more efficient. Another significant advantage was the reduction in sandbox sharing of resources and data, which streamlined the research process and reduced redundancies. Moreover, the ability to collect informed consent and PHI remotely and automatically through the development of scripts and Application Programming Interfaces (APIs) was a noteworthy advancement. This not only enhanced the efficiency of data collection but also aligned with the evolving needs of digital health research.

#### 2.2.2 Challenges

In Phase 2, while the transition to Azure improved certain aspects, several significant challenges persisted. Obtaining or producing high-quality, ongoing, or real-time datasets from Personal Health Information (PHI) remained a complex task. The IT management responsibilities, such as the development of scripts, APIs, and cloud-based pipelines for data transfer, continued to pose substantial barriers for public health researchers.

Furthermore, there were gaps in data governance frameworks, specifically in the alignment with standards like ISO/IEC 38500, as well as in cybersecurity standards and credential management. Another substantial challenge was the cost implications associated with each researcher establishing their resource group. This setup often involved unique virtual machines (VMs), storage accounts, Databricks instances, database servers, app services, and a variety of mostly underutilized resources. This not only led to inefficiencies but also contributed to increased costs.

In addition, there was limited IT infrastructure support for managing study participants' informed consent and re-consent processes, which is a crucial aspect of public health research. The cost management for processing the research data also remained inefficient, further complicating the overall effectiveness of the transition to the cloud-based environment.

### 2.3 Phase 3- UbiLab secure NIST environment

#### 2.3.1 Scenario and benefits

In Phase 3, the focus shifted to enhancing cybersecurity and data governance within the cloud environment to manage Personal Health Information (PHI) more effectively. This phase saw the incorporation of comprehensive security recommendations outlined in the National Institute of Standards and Technology (NIST) Special Publication 800–171. Additionally, it integrated compliance with multiple key regulatory frameworks, including Ontario's Freedom of Information and Protection of Privacy Act, the Personal Information Protection and Electronic Documents Act (PIPEDA), the General Data Protection Regulation (GDPR), the Personal Data Sovereignty Inter-Organizational Governance Framework for Public Health Research (43), and Azure's cloud governance framework. These integrations represented a significant advancement in the project's approach to data security and governance.

The introduction of these robust cybersecurity standards and data governance frameworks had a marked impact on enhancing the security and management of PHI. This development significantly improved trust with collaborators, as the enhanced security measures provided assurances for safer data exchanges. It also led to an increase in operational efficiency by effectively mitigating risks associated with unauthorized access. The alignment with international and regional data protection regulations further bolstered the framework's credibility and reliability, making it a more robust solution for managing sensitive health data.

#### 2.3.2 Challenges

In Phase 3, as the use of Azure increased, several new challenges emerged. The implementation of a virtual private network (VPN) for resource access became necessary, which in turn required the installation of a firewall and various security and performance applications, including Azure's NIST 800–171 blueprint initiative. This shift led to a significant escalation in the costs and complexity of managing networks, controlling user access, and configuring resources.

Additionally, public health researchers at UbiLab often lacked the necessary expertise to navigate these complex technical systems. This gap in knowledge necessitated one-on-one meetings to assist each researcher through the VPN setup process, as the existing documentation proved inadequate due to its technical jargon. The limited internet access from Azure resources further complicated matters, leading to stalled workflows and prolonged wait times for issue resolution.

Another challenge was the complexity involved in configuring and maintaining the resources deployed in Azure. Each new resource required extensive documentation and security measures such as firewall protection, logging, tagging, and password management. These tasks were often inadequately performed due to a shortage of human resources, which added to the challenges of maintaining a secure and efficient cloud-based environment.

### 2.4 Phase 4- secure UbiLab environment with a centralized data ecosystem

#### 2.4.1 Scenario and benefits

In Phase 4, the appointment of a dedicated cloud architect played a pivotal role. This specialist expedited the setup of VPNs and network configurations, significantly improving user support, resource configuration, and maintenance. Concurrently, there was a notable enhancement in cybersecurity measures. Additionally, a data governance program was established, featuring a committee composed of representative stakeholders. This committee was tasked with aligning UbiLab's data strategy with the internal objectives of stakeholders and developing a comprehensive data-sharing agreement.

The implementation of these measures in Phase 4 led to the creation of a secure, centralized cloud environment that is specifically designed for managing Personal Health Information (PHI) in public health research. A notable achievement during this phase was the reduction in Azure resource costs by ~30%−40%, which was primarily due to decreased data redundancy costs. Additionally, the establishment of the data governance program significantly streamlined the process of collecting data from data custodians, effectively reducing obstacles, and enhancing the efficiency of data management overall.

#### 2.4.2 Challenges

In Phase 4, the team faced a range of barriers related to data governance in healthcare, including concerns over user privacy, meeting data security requirements, setting appropriate data standards, and managing the intricacies of cross-institutional data collection and aggregation. The challenge of managing study participants' informed consent and the related costs was also significant.

To address these challenges, the team worked to establish semi-trusted relationships with stakeholders. This approach was supported by governance mechanisms such as clearly defined metrics, compliance monitoring, and auditing processes. These strategies were aimed at creating a robust and reliable framework for data governance, ensuring comprehensive management of all data aspects, from privacy to consent, in line with the broader objectives of the UbiLab project.

## 3 UbiSECE framework

The UbiLab Secure Cloud Environment (UbiSECE) was developed as the cumulative result of our experiential learning in PHI-based research (Phase 1–4). UbiSECE prioritizes data security; securely storing PHI data and providing controlled, role-based access defined by our cloud architect. Azure's governance functionalities enable us to define roles and responsibilities, monitor data usage and costs, and meet the traceability, accountability, auditability, and compliance needs of our stakeholders.

**Figure 1:**
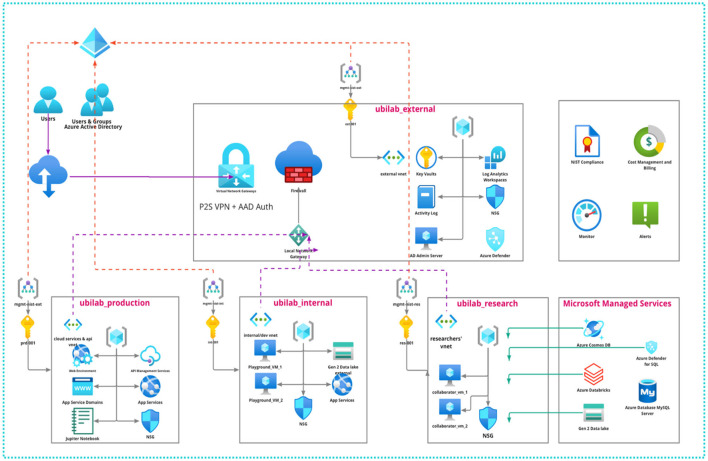
UbiSECE azure architecture for public health research.

UbiSECE's Azure Architecture comprises four main environments: UbiLab External, UbiLab Production, UbiLab Internal, and UbiLab Research (Figure 1).

*UbiLab_external*: This environment hosts resources, applications, APIs, or other services that are externally accessible without the need for a VPN and user account. It is designed with the highest degree of access flexibility in mind, allowing for wider data collection and interaction with external systems. However, given the open nature of this environment, no PHI is stored here. Any data collected in this environment via user interactions or APIs are transferred securely via Azure's private links to our secure data storage, thus maintaining the integrity and security of our data.

*UbiLab_production:* This domain hosts resources ready for production, serving as the active interface for deployed applications. It may include Python scripts collecting data from user sensors or a Jupyter notebook for a data science project shared with industry partners. This environment requires authentication and strict access control for any interaction. Only users with an Azure account, created and managed by our cloud administrator, can access these resources, ensuring that only authorized personnel can access these applications.

*UbiLab_internal*: This is a controlled environment where internal research projects are executed, hosted separately from the external and production domains. It's secluded from the Internet and does not involve industry partners. It offers collaborators controlled and cached access to portions of UbiLab's PHI data via virtual machines for research purposes. Direct access to centralized data storage is restricted, and any need for writing information into the central data storage requires specific privileges. As in the production environment, access requires passing through security layers and an Azure account created by our cloud administrator.

*UbiLab_research:* Dedicated to fostering academic research, this domain is exclusively reserved for UbiLab's Master's and Ph.D. students to conduct their thesis research. Although it shares the same restricted access controls as the internal and production environments, the UbiLab_research domain is distinct due to the nature of the work it hosts. It supports a wide range of academic activities, from experimental data science work to more structured, thesis-driven research projects. As in the other environments, access to resources in this domain is only possible through security layers and with an Azure account created by our cloud administrator.

## 4 Discussion

### 4.1 Strengths and scalability

Storing and managing Personal Health Information (PHI) is a major challenge in public health research. Here we outlined the progress toward the development of UbiSECE: a private and secure cloud-based data governance framework. UbiSECE employs role-based access controls to centralized data storage to ensure the security of PHI while enabling public health research.

One of the key strengths of our cloud-based solution is its scalability and accessibility. UbiSECE allows public health researchers to store and analyze large volumes of data efficiently and facilitates seamless collaboration among different teams. The UbiSECE framework also paves the way for future integration with PHR systems, enabling seamless sharing and utilization of medical records for research purposes. The scalability of the UbiSECE framework is twofold, encompassing both vertical and horizontal dimensions. Vertically, it can expand its capacity to accommodate larger datasets and more complex processing needs. Horizontally, the framework is designed to integrate emerging technologies and adapt to new research demands, ensuring its utility in the evolving landscape of public health research.

Another strength lies in the framework's compliance with healthcare standards and regulations including NIST 800–53, PIPEDA, HIPAA, and GDPR. The framework ensures that PHI is handled according to established security protocols and sets a high standard for ethical and responsible data governance. Looking forward, UbiSECE is strategically positioned to evolve with the advancements in technology and the increasing demands for data in public health research. Its design and infrastructure are geared toward adaptability and scalability, ensuring its relevance and efficacy in the future.

### 4.2 Challenges in data access and security

Despite these benefits, managing data access for new collaborators or researchers remains complex. Currently, access is granted by cloud administrators through registered user accounts with limited privileges. Streamlining and automating this process could enhance collaboration and expedite research activities. Furthermore, although the framework ensures data security, ongoing efforts are needed to refine governance programs and fully comply with NIST-800–171 and NIST-800–52 standards. Continuous improvement and regular audits are essential to mitigate emerging cybersecurity threats and maintain the integrity of the cloud infrastructure.

### 4.3 Evaluation and feedback

In recognizing the importance of continuous improvement, our framework includes robust evaluation and feedback mechanisms. Weekly leadership meetings are conducted with researchers to discuss the functioning and efficacy of the UbiSECE framework. These meetings serve as a platform for researchers to provide feedback on their experiences, challenges faced, and suggestions for improvements. Adjustments to the system and processes are made as needed, based on this feedback. Additionally, monthly meetings are held with stakeholders to ensure their perspectives and requirements are effectively integrated into the framework. This iterative process of gathering and implementing feedback ensures that the UbiSECE framework remains responsive to the needs of its users and up to date with the latest developments in public health research.

### 4.4 Training and user support

UbiLab's transition to the UbiSECE framework is supported by training sessions conducted by our dedicated cloud architect. These targeted one-on-one sessions equip researchers with the necessary skills to navigate and utilize the cloud-based system effectively. These sessions cover a range of topics, from basic navigation of the Azure cloud environment to advanced data management and security protocols. Additionally, comprehensive user guide to provide ongoing support and address common technical queries were provided to the researchers.

### 4.5 Practical applications

In the context of UbiLab's current projects (44–52), the UbiSECE framework is actively employed in a variety of research areas, demonstrating its practicality and versatility. These initiatives include using IoT for monitoring climate change behaviors and chronic disease risks (45, 47), analyzing big data for public health studies on air pollution effects (51), and applying smart home technologies for older adult healthcare (52). This range of applications highlights UbiSECE's effectiveness in enhancing both research efficiency and data security, showing its potential as a key tool in public health research.

### 4.6 Ethical considerations

The transition to cloud-based systems for managing Personal Health Information (PHI) necessitates a comprehensive examination of ethical considerations that extend beyond informed consent. The adoption of cloud computing in healthcare brings to the fore critical questions regarding data ownership, patient confidentiality, and the potential for data misuse (53). To ensure patient confidentiality within cloud environments, robust encryption, and sophisticated access control mechanisms must be employed, alongside clear policies on data ownership that honor patient rights and adhere to legal standards. Moreover, the risk of data misuse—whether by intent or accident—necessitates the implementation of stringent governance frameworks and the conduction of regular audits. These steps are imperative to uphold compliance with ethical standards and legal requirements. Addressing these ethical dimensions is crucial to maintain trust in cloud-based healthcare systems and to safeguard the integrity of PHI.

### 4.7 Future directions and cost-effectiveness

Future research should explore advanced data analytics techniques and machine learning algorithms within the cloud-based framework to extract valuable insights from healthcare data. Azure's machine learning capabilities could be leveraged to develop predictive models and decision support systems for public health research. Investigating the interoperability and data exchange standards between different cloud platforms and PHR systems could facilitate data sharing and collaboration. Finally, continuous evaluation of the framework's performance and security measures and monitoring of emerging healthcare regulations and standards will ensure its effectiveness and adaptability in an evolving healthcare landscape.

Additionally, it's pertinent to note the financial aspects of the UbiSECE framework implementation. Initially, UbiLab incurred upfront costs for data migration, staff training, and system setup in adopting cloud technology. However, these were effectively balanced by long-term savings, including a ~30%−40% reduction in Azure resource costs, primarily due to decreased data redundancy and enhanced operational efficiencies. The scalability of cloud solutions also mitigated the need for substantial future investments in IT infrastructure, further underscoring the cost-effectiveness of this transition.

The frameworks developed here can support interdisciplinary research and accelerate knowledge discovery while safeguarding public health information.

## Author contributions

PMo: Project administration, Resources, Supervision. JK: Writing—original draft, Conceptualization, Methodology, Visualization, Writing—review & editing. PMi: Writing—original draft, Data curation, Methodology, Visualization, Writing—review & editing.

## Glossary

Cloud Technology: Online computing services for data storage and processing.

PHI (Personal Health Information): Identifiable health and healthcare payment data of individuals.

Azure: A cloud computing service by Microsoft for app services and data management.

NIST 800-53: U.S. standards for information security in federal systems.

GDPR (General Data Protection Regulation): EU law for data protection and privacy.

RBAC (Role-Based Access Control): A system of managing user access based on roles.

HIPAA (Health Insurance Portability and Accountability Act): U.S. law for medical information privacy.

PIPEDA (Personal Information Protection and Electronic Documents Act): Canadian data privacy law for commercial sectors.

API (Application Programming Interface): Rules for software components interaction.

VPN (Virtual Private Network): A secure network connection over the internet.

NIST 800-171: U.S. guidelines for protecting non-classified information.

Data Governance: Management of data availability, usability, integrity, and security.

Cybersecurity: Protection of systems and networks from digital attacks.

Machine Learning: AI that enables software to predict outcomes more accurately.

Data Analytics: Analyzing raw data to find trends and insights.
